# Author’s response: influenza-associated mortality oseltamivir: beware of misstepping into stepwise procedures

**DOI:** 10.2807/1560-7917.ES.2019.24.46.1900692

**Published:** 2019-11-14

**Authors:** Mark Reacher, Neville Q. Verlander, Maria Zambon, on behalf of the authors of the original article

**Affiliations:** 1 Public Health England Field Service, Cambridge Institute of Public Health, Cambridge, United Kingdom; 2 Public Health England and Cambridge Universities Hospitals NHS Foundation Trust Cambridge, Cambridge, United Kingdom; 3 Statistics Unit, Statistics, Modelling and Economics Department, National Infection Service – Data and Analytical Sciences, Public Health England, London, United Kingdom; 4 National Infection Service, Public Health England, London, United Kingdom; 5 The authors are acknowledged at the end of the article

We thank Lytras et al. [1] for the comments on our recent article assessing risk factors for mortality in inpatients with influenza and the effect of oseltamivir [2]. For this study, the authors are confident that the identification and adjustment for confounding variables have been done in a systematic and objective manner using stepwise logistic regression. We note the concerns on the methods we have used, but we do not see how these concerns are specific to our work. Rather the concerns relate in general to the application of these methods.

We observed evidence of a protective association of full course 5 days treatment with oseltamivir against inpatient mortality, which emerged following adjustment for the confounding variables we identified. We believe this is plausible, given that risk of death is widely recognised to be associated with comorbidities and age, and that oseltamivir is licensed as an effective medication for seasonal influenza with a rational drug design against a viral target.

It is the case that multiple testing using regression analysis has occurred and the authors agree that accounting for this could result in an upper limit exceeding 1. However, it would still be the case that evidence of an association between standard course of oseltamivir and protection against inpatient mortality was demonstrated in a relatively large historic cohort of hospitalised patients receiving routine clinical care.

Lytras et al. [1] write that the final multivariable model contained nine predictors in a dataset with just 32 outcome events (deaths), that our model was severely overfitted suggesting that the low prevalence of several risk factors in the data hints at potential multicollinearity problems and that, both during the stepwise procedure and in the final model, Variance Inflation Factors for the covariates should have been included (i.e. in Table 2 of the article [2]). In response, we note there were only seven variables in the final model of Table 2 with a total of nine parameters and do not agree that there is universal acceptance of the 10 events per variable recommendation [3]. In any situation, the required number of observations could be more or less than this number. An over fitted model would be manifested in extreme estimates and confidence intervals. All the estimates and confidence intervals presented are proportionate, with the wide confidence interval for ‘excessive alcohol use’ reflecting the few individuals with that factor. The nonlinear link function used in the logistic regression has meant that collinearity would have needed to have been severe for it to have caused difficulties, which would have been manifested in the estimates and confidence intervals.

With regard to delay in Table 3 of our study [2], for those not receiving oseltamivir, the delay was coded as zero. A binary variable defined to be one if antiviral given and zero otherwise was created in order to assess the impact of delay on mortality by interacting it with delay whenever delay was to be analysed.
